# Localization of Melanocortin 1 Receptor in the Substantia Nigra

**DOI:** 10.3390/ijms26010236

**Published:** 2024-12-30

**Authors:** Ayuka Ehara, Nozomi Ito, Kazuhiko Nakadate, Nobuko Tokuda

**Affiliations:** 1 Department of Anatomy, Dokkyo Medical University School of Medicine, 880 Kita-Kobayashi, Mibu-machi, Shimotsuga-gun 321-0293, Tochigi, Japan; tokudan@dokkyomed.ac.jp; 2 Department of Functional Morphology, Meiji Pharmaceutical University, 2-522-1 Noshio, Kiyose 204-8588, Tokyo, Japan; s212009@std.my-pharm.ac.jp (N.I.); nakadate@my-pharm.ac.jp (K.N.)

**Keywords:** melanocortin 1 receptor, attractin, dopamine, parvalbumin, mitochondria

## Abstract

Recent findings have revealed that melanocortin 1 receptor (MC1R) deficiency leads to Parkinson’s disease-like dopaminergic neurodegeneration in the substantia nigra (SN). However, its precise distribution and expressing-cell type in the SN remain unclear. Therefore, in this study, we analyzed the localization and characteristics of MC1R in the SN using histological methods, including in situ hybridization and immunohistochemistry. Our findings reveal that MC1R was slightly present in dopaminergic neurons in the ventral tier of SN pars compacta dorsal (vSNCD), a region particularly vulnerable to PD-related neurodegeneration. Notably, we discovered that MC1R is highly present in parvalbumin (PV)-positive neurons, which were also *vesicular GABA transporter* messenger RNA-expressing inhibitory neurons of the lateral SN pars reticulata (lSNR). Intracellular analysis demonstrated that MC1R was present not only in the plasma membrane but also in mitochondrial and endoplasmic reticulum membranes. Furthermore, MC1R co-localized with attractin (Atrn), a known MC1R modulator, in nearly all MC1R-positive neurons. Therefore, it has been suggested that MC1R and Atrn work together to regulate dopaminergic neurons in the SN through both direct expression and indirect modulation via PV-positive inhibitory neurons. These findings provide new insights into MC1R’s role in the SN and its potential contribution to PD pathophysiology

## 1. Introduction

The melanocortin 1 receptor (MC1R) is a cyclic AMP-stimulating G protein-coupled receptor that regulates skin physiology through the melanin synthesis pathway. The *MC1R* gene is the main pigmentation gene, and its loss-of-function variants are associated with the risk of developing melanoma [1]. Recent epidemiological studies have suggested the relative risk of malignant melanoma and Parkinson’s disease (PD) [2,3,4]. PD is a progressive neurodegenerative disease of the central nervous system, and dopaminergic (DA) neurons in the substantia nigra (SN) gradually degenerate. Notably, MC1R is reportedly present not only in skin melanocytes but also in neurons and glial cells in the brain [5,6]. In parallel with these clinical data, basic research is ongoing to analyze MC1R function using animal models of DA neurodegeneration, including PD models [7,8,9]. In MC1R nonfunctioning mice (MC1R^e/e^ mice), DA neurons are more susceptible to degeneration with age, and increased sensitivity to 6-hydroxy dopamine and 1-methyl-4-phenyl-1,2,3,6-tetrahydropyridine leads to progressive degeneration of DA neurons [7]. Furthermore, the activation of MC1R using specific agonists reduces alpha-synuclein aggregation and inflammation, which are critical pathological features of PD [8]. These results suggest that MC1R protects DA neurons from degeneration by modulating inflammatory responses and preventing the accumulation of toxic proteins in these neurons. However, the distribution of MC1R-positive cells in the SN, where DA neurons degenerate in PD cases, has not been analyzed in detail. Notably, some reports indicate that MC1R is prominent in DA neurons of the SN, whereas others report that it is only faintly observed [7,8,10]. The SN also contains non-DA neurons, some of which regulate DA neuronal function. Therefore, to accurately understand the function of MC1R, it is necessary to identify the distribution of MC1R and the cell types that express it.

Attractin (Atrn) modulates the interplay between MC1R and its ligand, agouti signaling protein, which regulates pheomelanin synthesis in skin melanocytes [11]. Furthermore, Atrn is present in the DA neurons of the SN in the central nervous system, and its deletion leads to the progression of dopaminergic neurodegeneration [12,13,14,15,16]. Therefore, MC1R may coexist and cooperate with Atrn in the SN.

We hypothesized that MC1R and Atrn coexist and cooperate in the dopamine neurons and their regulatory neurons of the SN. Consequently, in this study, to clarify the localization of MC1R in the SN, we first examined the distribution of *MC1R* messenger RNA (mRNA)-expressing cells using two in situ hybridization methods and the distribution of MC1R-positive cells using immunostaining. In addition, cell types, including dopaminergic neurons in which MC1R is present, were identified. Furthermore, we examined whether MC1R coexists with Atrn in the SN.

## 2. Results

### 2.1. Distribution of MC1R mRNA-Expressing Cells in the SN

**Figure 1:**
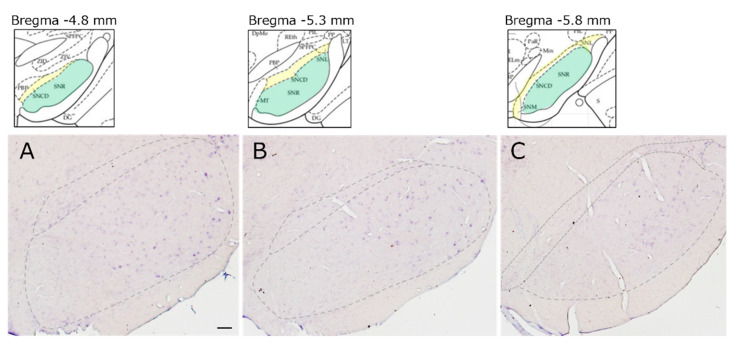
Distribution of *MC1R* mRNA-expressing cells in the SN. Distribution of DIG-probed *MC1R* mRNA-labeled cells on representative coronal sections in the substantia nigra (SN). Level of bregma: (**A**) −4.8 mm, (**B**) −5.3 mm, and (**C**) −5.8 mm. Scale bar: 100 µm.

In situ hybridization performed on sections of the SN using digoxigenin (DIG)-labeled probes revealed the distribution of *MC1R* mRNA-expressing cells (Figure 1). *MC1R* expression was strong in the lateral SN pars reticulata (lSNR) and lateral SN (SNL), it was but weak in SN pars compacta dorsal (SNCD). Notably, in the SNCD, cells faintly expressing *MC1R* were observed at the ventral tier of SN pars compacta dorsal (vSNCD).

### 2.2. Identification of MC1R-Expressing Cell Types in the SN

**Figure 2:**
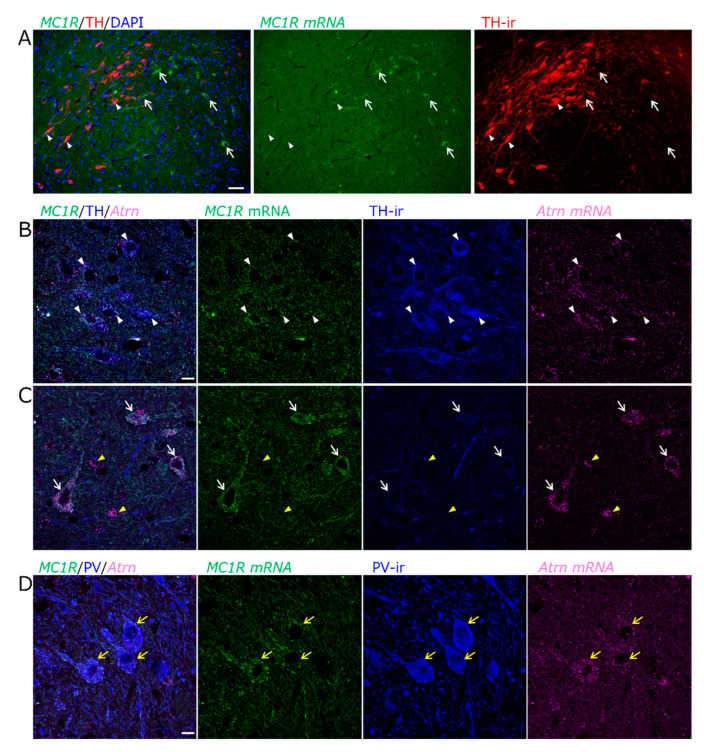
Weak *MC1R* expression in TH^+^ dopaminergic neurons of the SN. (**A**) Epifluorescence images for *MC1R* mRNA (green), TH immunoreactivity (TH-ir, red), and counterstained with DAPI (blue) in the SN. Expression of *MC1R* in TH^+^ dopaminergic neurons of the ventral tier of SN pars compacta dorsal (vSNCD, arrowheads) or TH^−^ cells of the lateral SN pars reticulata (lSNR, arrows). Scale bar: 50 µm. (**B**,**C**) Confocal microscopy images for *MC1R* mRNA (green), TH-ir (blue), and *Atrn* mRNA (magenta) in the SN. (**B**) TH^+^ neurons weakly express *MC1R* and co-express *Atrn* in the vSNCD (white arrowheads). (**C**) Some TH^−^ cells strongly express *MC1R* and co-express *Atrn* in the lSNR (white arrows). TH^−^ small cells that do not express *MC1R* include cells that express only *Atrn* (yellow arrowheads). Scale bar: 10 µm. (**D**) Confocal microscopy images for *MC1R* mRNA (green), PV-ir (blue), and *Atrn* mRNA (magenta) in the lSNR. *MC1R* signals are strong in PV^+^ neurons, and *Atrn* signals are also strong (yellow arrows). Scale bar: 10 µm.

A combination of in situ hybridization chain reaction (HCR) and immunofluorescence staining was used to identify cell types expressing *MC1R*. The distribution of *MC1R* mRNA-expressing cells resulting from in situ HCR was similar to that observed using the DIG-labeled probes (Figure 2A). *MC1R* was slightly expressed in some tyrosine hydroxylase (TH)-positive dopaminergic neurons in the vSNCD (Figure 2A,B). In contrast, *MC1R* was prominently expressed in TH-negative cells of the SNR (Figure 2A,C). lSNR is a region where parvalbumin (PV) neurons are prominent; therefore, we examined whether TH-negative cells with intense *MC1R* expression were PV-positive neurons. In the lSNR, intense *MC1R* expression was observed in the PV-positive neurons (Figure 2D). Furthermore, most cells expressing *MC1R* also co-expressed *Atrn* (Figure 2B,C). Some cells that did not express *MC1R* expressed only *Atrn* (Figure 2C).

**Figure 3:**
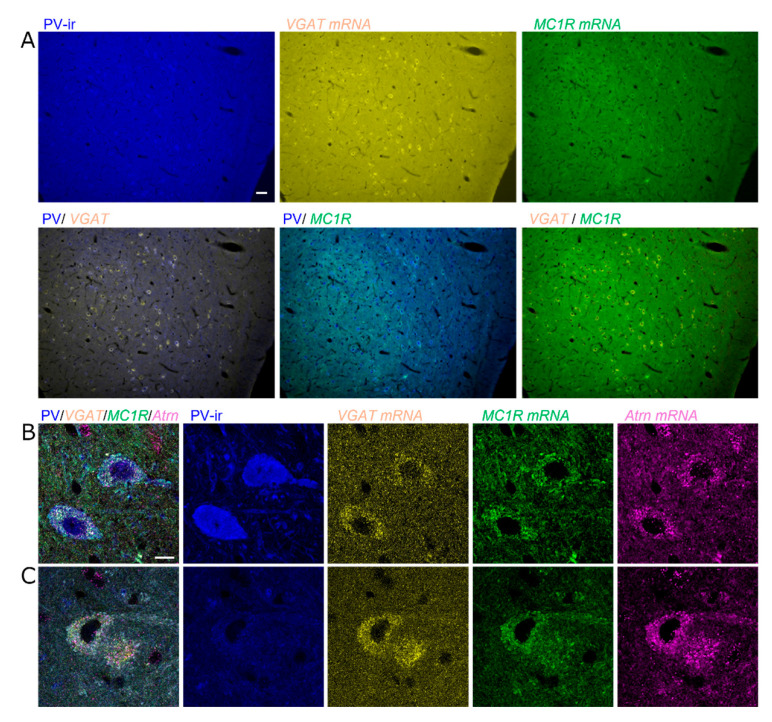
Strong *MC1R* expression in PV^+^ or PV^−^ GABAergic neurons marked with *VGAT* of the SNR. (**A**) Epifluorescence images for PV-ir (blue), *VGAT* mRNA (yellow), and *MC1R* mRNA (green) in the lSNR. The top column shows individual images, and the bottom column shows the combinations of two images for each image: PV-ir/*VGAT* mRNA, PV-ir/*MC1R* mRNA, and *VGAT* mRNA/*MC1R* mRNA. Scale bar: 50 µm. (**B**,**C**) Confocal microscopy high-magnified images for PV-ir (blue), *VGAT* mRNA (yellow), *MC1R* mRNA (green), and *Atrn* mRNA (magenta) in the lSNR. Scale bar: 10 µm. (**B**) PV^+^ GABAergic neurons marked with *VGAT* strongly express both *MC1R* and *Atrn*. (**C**) Even some PV^−^ GABAergic neurons marked with *VGAT* strongly express both *MC1R* and *Atrn*.

PV neurons in the lSNR are known as GABAergic inhibitory neurons. However, some GABAergic inhibitory neurons in this region are negative for PV. Therefore, we examined whether *MC1R* is expressed in GABAergic inhibitory neurons, including PV neurons. We verified their GABAergic identity by assessing the expression of the *vesicular GABA transporter* (*VGAT*). *MC1R* was present in PV-positive and PV-negative GABAergic inhibitory neurons expressing *VGAT* in the lSNR (Figure 3A,B). However, *MC1R* was not expressed in *VGAT*-expressing GABAergic neurons in SNR regions other than the lateral regions (Figure 3A). Notably, both types of *MC1R*-expressing neurons expressed *Atrn* (Figure 3B,C). These findings indicated that *MC1R* expression was more prominent in inhibitory PV neurons than in dopaminergic neurons in the SN. We also found that *Atrn* was co-expressed in almost all types of neurons in which *MC1R* was expressed.

### 2.3. Distribution of MC1R Immunopositive Cells in the SN

**Figure 4:**
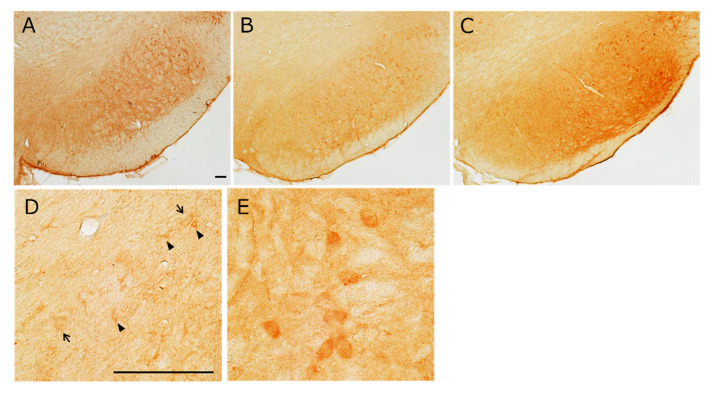
Distribution of MC1R^+^ cells in the SN. Distribution of DAB-amplified MC1R^+^ cells on representative coronal sections of the SN. Level of bregma: (**A**) −4.8 mm, (**B**) −5.3 mm, and (**C**) −5.8 mm. (**D**) Magnified MC1R-ir images of SNCD. MC1R immunoreactivity is weak in large neuron-like cells (arrows) and strong in small cells (arrowheads) of the vSNCD. (**E**) Magnified MC1R-ir images of lSNR. MC1R immunoreactivity is very strong in large neuron-like cells. Scale bar: 100 µm.

Furthermore, to determine whether *MC1R* mRNA-expressing corresponds to MC1R protein synthesis, immunohistochemistry for MC1R was performed. The distribution of MC1R-positive cells was similar to that of *MC1R*-expressing cells (Figure 4A–E). Additionally, MC1R immunoreactivity was weak in the larger cells and strong in the smaller cells of the vSNCD (Figure 4D). In contrast, its immunoreactivity was very strong in the large cells of the lSNR (Figure 4E).

### 2.4. Identification of MC1R Immunopositive Cell Types in the SN

**Figure 5:**
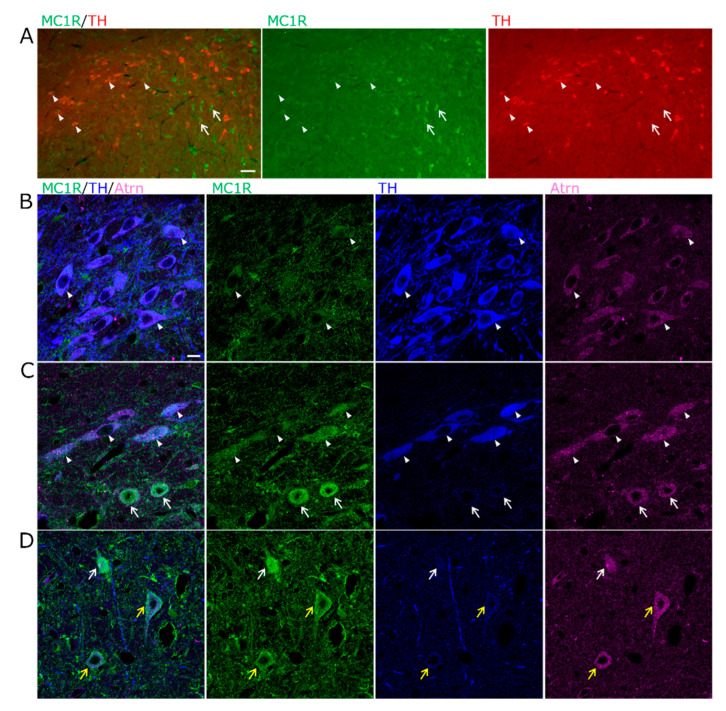
Co-localization of MC1R and Atrn in TH^+^ dopaminergic neurons of the SN. (**A**) Epifluorescence images for MC1R (green) and TH (red) in the SN. MC1R is faintly present in some TH^+^ dopaminergic neurons of the vSNCD (arrowheads). In the TH^−^ cells of the SNL and the lSNR, MC1R immunofluorescence is extremely strong (arrows). Scale bar: 50 µm. (**B**–**D**) Confocal microscopy high-magnified images for MC1R (green), TH (blue), and Atrn (magenta) in (**B**,**C**) the lateral vSNCD, or (**D**) the lSNR. In some TH^+^ dopaminergic neurons of the vSNCD, MC1R is faintly present throughout the cytoplasm, and Atrn also coexists in dots (arrowheads). In some TH^−^ cells of the vSNCD and lSNR, MC1R strongly localizes to the cytoplasm (white arrows) and plasma membrane (yellow arrows), with Atrn also coexisting in dots. Scale bar: 10 µm.

**Figure 6:**
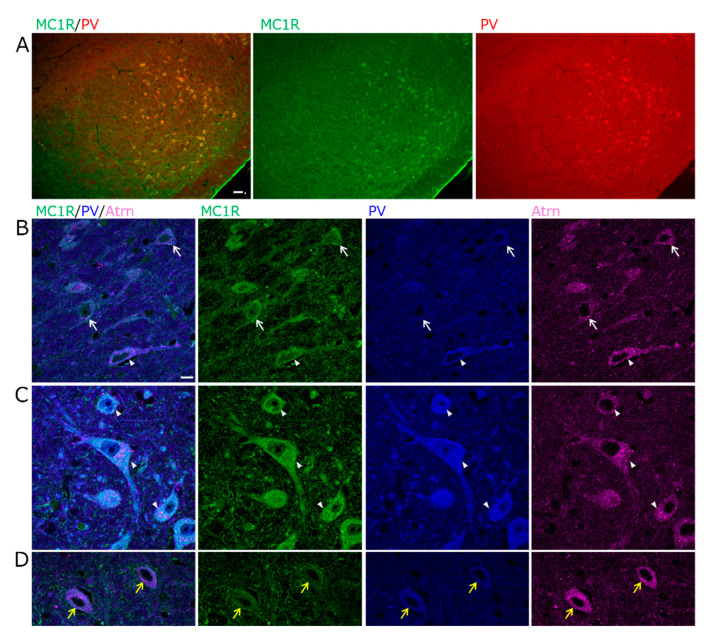
Co-localization of MC1R and Atrn in PV^+^ neurons of the SN. (**A**) Epifluorescence images for MC1R (green) and PV (red) in the SNR. MC1R is present in PV^+^ neurons of the lSNR, and its immunofluorescence is extremely strong. Scale bar: 50 µm. (**B**–**D**) Confocal microscopy high-magnified images for MC1R (green), PV (blue), and Atrn (magenta) in (**B**) the vSNCD or (**C**,**D**) the lSNR. In PV^+^ neurons, MC1R is predominantly localized in the cytoplasm and Atrn and found in dot-like structures (white arrowheads). In PV^−^ cells of the SNCD, MC1R is faintly present throughout the cytoplasm, and Atrn is also coexistent (white arrows). In some PV^−^ cells with Atrn of the SNR, MC1R is localized at the plasma membrane (yellow arrows). Scale bar: 10 µm.

Multifluorescent labeling was performed to identify MC1R-positive cell types. MC1R was faintly present throughout the cytoplasm in TH-positive dopaminergic neurons of vSNCD (Figure 5A–C). MC1R was also localized in the TH-negative cells of the SNL and lSNR, and its immunofluorescence was extremely strong (Figure 5A,C,D). In these cells, MC1R was present throughout the cytoplasm (Figure 5C,D) or localized to the plasma membrane (Figure 5D). Notably, some MC1R-positive SNL and lSNR cells were PV-positive neurons (Figure 6A). MC1R was present throughout the cytoplasm of these PV neurons, and its immunofluorescence was extremely strong (Figure 6C). Furthermore, the PV-negative cells of the vSNCD, in which the cells may be dopaminergic neurons, had MC1R in the cytoplasm (Figure 6B). Conversely, those of the lSNR also had MC1R in the plasma membrane (Figure 6D).

**Figure 7:**
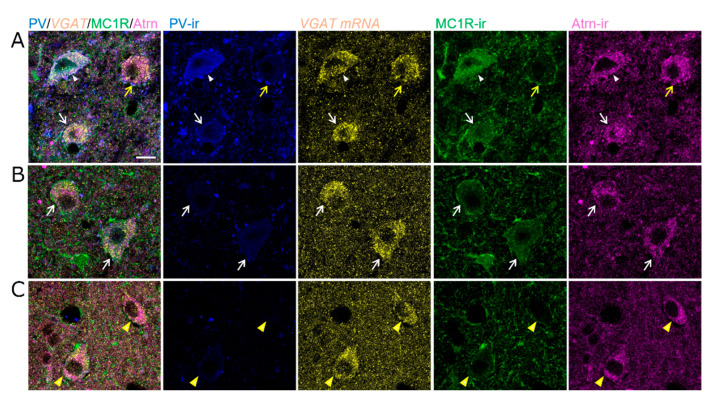
Co-localization of PV, MC1R, and Atrn in *VGAT* expressing GABAergic neurons of the SNR. High-magnification confocal microscopy images of PV (blue), *VGAT* mRNA (yellow), MC1R (green), and Atrn (magenta) in (**A**) dorsolateral, (**B**) medial, and (**C**) ventral SNR. PV^+^ neurons are labeled with *VGAT*, MC1R, and Atrn (white arrowheads). GABAergic neurons identified by *VGAT* without PV are of two types: those with MC1R and Atrn (white arrows) and those with only Atrn but no MC1R (yellow arrows and arrowheads). In GABAergic neurons co-labeled with MC1R and Atrn, MC1R is faintly present in the cytoplasm and is strongly localized to the plasma membrane (white arrows). GABAergic neurons with only Atrn are surrounded by PV^+^ (yellow arrows) or PV^−^ (yellow arrowheads) processes with MC1R. Scale bar: 10 µm.

In the SNR, PV-positive and PV-negative GABAergic inhibitory neurons express *VGAT*. Notably, PV-negative *VGAT*-expressing neurons were smaller than PV-positive neurons. Furthermore, PV-negative *VGAT*-expressing neurons included neurons with and without MC1R in the mediolateral and ventral SNR, respectively. In *VGAT*-expressing neurons with MC1R, MC1R was slightly stained in the cytoplasm and prominently localized to the plasma membrane (Figure 7A,B). Notably, some *VGAT*-expressing neurons without MC1R were surrounded by MC1R-positive processes, and two types of MC1R-positive processes were observed: PV-positive and PV-negative (Figure 7A,C).

The examination of co-localization of MC1R and Atrn revealed that almost all MC1R-positive neurons were simultaneously positive for Atrn (Figure 5B–D, Figure 6B–D and Figure 7A). Atrn was also observed in GABAergic neurons lacking MC1R (Figure 7A,C).

### 2.5. Intracellular Location of MC1R

**Figure 8:**
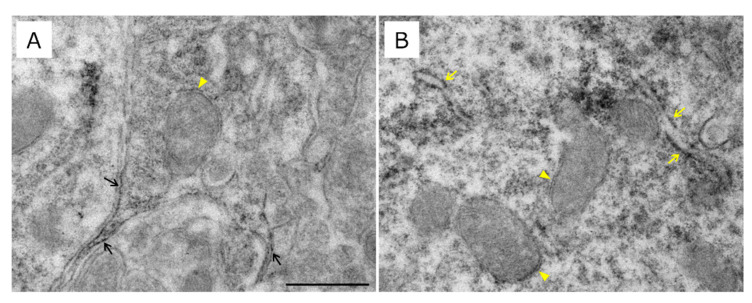
Intracellular localization of MC1R in the lSNR. (**A**,**B**) Immunoelectron micrograph of MC1R in the cell body of the lSNR. MC1R is located on the cell membrane (black arrows), endoplasmic reticulum membrane (yellow arrows), and mitochondrial membrane (yellow arrowheads). Scale bar: 0.5 µm.

Finally, immunoelectron microscopy was performed to examine the intracellular localization of MC1R (Figure 8). MC1R was present in the plasma membrane and the mitochondrial (Figure 8A,B) and endoplasmic reticulum (Figure 8B) membranes. Furthermore, MC1R was observed throughout the cytoplasm (Figure 8B).

## 3. Discussion

This is the first detailed histological analysis of the localization of MC1R in the SN. TH-positive dopaminergic neurons in the vSNCD showed *MC1R*-mRNA expression and MC1R-positive immunoreactivity; however, both responses were weak. MC1R is reportedly present in dopaminergic neurons in the SN; however, immunostaining results have been inconsistent, with some reports showing very strong staining and others showing very weak staining [7,8,10]. In this study, we accurately identified MC1R-immunopositive neurons by examining the distribution of these positive neurons alongside mRNA-expressing neurons and ensuring consistency in the intensity between the two. The distribution of *MC1R* mRNA-expressing cells was confirmed using two different in situ hybridization methods: the DIG method, using full-length RNA probes; and the HCR amplification method, using multiple DNA probes for the target mRNA. Thus, the results of MC1R immunostaining in this study are highly credible.

Notably, various subgroups of dopaminergic neurons exist in the SN, and those in the vSNCD are particularly susceptible to neurodegeneration in PD cases [17]. Recent studies have identified a specific subtype of vulnerable neurons that expresses cytoplasmic aldehyde dehydrogenase 1 (Aldh1) and G protein-activated inwardly rectifying potassium type 2 (Girk2) in the vSNCD [18,19,20,21]. Therefore, a reduction in the levels of these proteins is correlated with dopaminergic neurodegeneration in PD cases. In this study, dopaminergic neurons faintly stained for MC1R were found exclusively in the vSNCD. Thus, these MC1R neurons may be members of this vulnerable dopaminergic neuron subtype.

Surprisingly, *MC1R* was strongly expressed in non-dopaminergic neurons of the SN. These cells were PV-positive neurons and *VGAT* mRNA-expressing neurons in the SNR. MC1R exhibited the strongest mRNA expression in PV-positive neurons of the lSNR and displayed strong immunoreactivity at the plasma membrane and intracellularly (Supplemental Materials Figure S1). Immunoelectron microscopy images of MC1R demonstrated robust immunoreactivity in the rough endoplasmic reticulum and plasma membrane. These findings suggest that a significant amount of MC1R is synthesized in PV-positive neurons and supplied to the plasma membrane. Notably, VGAT marks functional GABAergic neurons, signaling that GABA is ready for synaptic release [22,23]. Therefore, we identified the *VGAT* mRNA expression site as the cytoplasm of GABAergic neurons. In this study, PV-positive neurons also expressed *VGAT* mRNA, consistent with previous findings that PV-positive neurons are GABAergic inhibitory neurons [24,25]. Projections from PV neurons in the SNR to the SNCD effectively inhibit dopaminergic neurons [24,25,26]. These results suggest that MC1R is involved in the inhibitory function of dopaminergic neurons in the SNCD through PV neurons in the SNR. PV neurons exhibit increased firing rates during high motor activity, and their activation facilitates movement termination, aligning with the role of the SN in inhibiting unwanted movement during action selection [27]. Moreover, PV neurons are crucial in exerting an immediate locomotor drive through the modulation of SNCD dopaminergic activity [25]. MC1R may also be involved in these processes. The dysfunction of the *MC1R* gene has been shown to significantly worsen the severity of dopaminergic neurodegeneration in the SN associated with Parkinson’s disease [8,28]. Thus, MC1R dysfunction promotes neurodegeneration and disrupts the critical inhibitory role of dopaminergic neurons, a process that may be intricately associated with PV neurons.

*VGAT* is moderately expressed in PV-positive neurons and strongly expressed in PV-negative neurons, typical GABAergic neurons. Therefore, the presence of MC1R in PV-negative GABAergic neurons is inconsistent. Notably, a few cells in the lSNR group demonstrated the presence of the MC1R; however, most neurons in the vSNR group were devoid of this receptor. When MC1R is present, it is primarily located in the plasma membrane with minimal localization in the cytoplasm. This suggests that MC1R is significant in the membrane-associated functions of GABAergic neurons in the lSNR. In addition to the PV subset, GABAergic neurons in the SNR have been recognized for their role in maintaining continuity in motor patterns by modulating thalamocortical activity or promoting quiescence through the suppression of motor activity and brain arousal [25,27]. Therefore, given these findings, the GABAergic neurons of the SNR can be further classified into ventral and lateral GABAergic neurons by combining the presence of MC1R with the presence of PV. This classification may be important in understanding the distinct functional roles of GABAergic neurons.

Our results showed that almost all MC1R-positive neurons in the SN contain Atrn. In dopaminergic, PV, and GABAergic neurons, MC1R was found in the cytoplasm, cytoplasm and plasma membrane, and plasma membrane, respectively. In contrast, Atrn was predominantly located in the cytoplasm of these cell types; however, some images appeared adjacent to MC1R. Atrn may act as a modulator of MC1R in the SN, as well as in the skin. Recent studies suggested that MC1R activates antioxidant pathways against oxidative stress and protects mitochondrial biogenesis and function [29,30,31]. In addition, Atrn has been suggested to exert neuroprotective effects against oxidative stress [32,33,34,35,36] and is localized in the mitochondrial membrane [15,35]. In this study, we found that MC1R was also present in the mitochondrial membrane. Therefore, MC1R and Atrn may interact with the mitochondrial membrane to provide neuroprotection against oxidative stress.

## 4. Materials and Methods

### 4.1. Animals

Male Sprague Dawley rats (SD rats, Japan SLC, Shizuoka, Japan), aged 2–3 months, were used. Notably, all animals were housed in groups of two or three per cage with continuous access to food and water and maintained on a 12 h light/dark cycle at the Laboratory Animal Research Center at Dokkyo Medical University School of Medicine. Furthermore, all procedures in this study were certified by the Dokkyo University School of Medicine Animal Welfare Committee (Permission number: 0570) and were performed according to the NIH guidelines.

### 4.2. In Situ Hybridization

Animals were anesthetized with a combination anesthetic (0.4 mg/kg of medetomidine, 2.0 mg/kg of midazolam, and 2.5 mg/kg of butorphanol) and then transcardially perfused with physiological saline, followed by an ice-cold fixative containing 4% paraformaldehyde in 0.1 M phosphate-buffered (PB, pH 7.4). The brains were postfixed in a fresh fixative overnight at 4 °C, infiltrated with 30% sucrose for 2 days at 4 °C, embedded for cryo-sectioning in Tissue-Tek OCT (Sakura Finetek Japan Co., Tokyo, Japan), frozen with powdered dry ice, and sectioned at 16 µm on a Cryostat NX70 (Thermo Fisher Scientific K.K., Tokyo, Japan). The sections in antifreeze solution (30% ethylene glycol-glycerol in 0.1 M phosphate-buffered saline: PBS) were stored at −20 °C until hybridization. Sections were incubated for 10 min in 0.1 M PB containing 0.5% Triton X-100, treated with 0.2 N HCl for 15 min, and then acetylated in 0.25% acetic anhydride in 0.1 M triethanolamine (pH 8.0) for 10 min. Sections were postfixed in 4% paraformaldehyde for 15 min. Before hybridization, free-floating sections were incubated in hybridization buffer (50% formamide; 10% dextran sulfate; 5× Denhardt’s solution; 620 mM NaCl; 50 mM dithiothreitol; 10 mM Ethylenediaminetetraacetic acid (EDTA); 20 mM Piperazine (pH 6.8); 0.2% sodium dodecyl sulfate; 250 μg/mL transfer RNA; and 250 μg/mL salmon sperm DNA) for 2 h at 55 °C. DIG-labeled RNA probes were generated using pBluescript II SK(+) with the rat MC1R gene (963 bp, XM_006255795.3) inserted into the HindIII/BamHI sites (GenScript, Tokyo, Japan). Antisense and sense probes were prepared using T3 and T7 RNA polymerases. Sections were hybridized for 18 h at 55 °C in a hybridization buffer containing a mixture of the DIG-labeled MC1R probe. Sections were washed with 2× saline sodium citrate (SSC); treated with 5 μg/mL RNase A at 37 °C for 1 h; washed with 0.5 × SSC, 50% formamide, 0.5% sarkosyl, and 10 mM beta-mercaptoethanol (beta-ME) at 55 °C for 1 h 30 min; and then washed with 0.1 × SSC, 0.5% sarkosyl and 10 mM beta-ME at 60 °C for 1 h. After the last 0.1 × SSC wash, the sections were rinsed with PBS. Furthermore, after treatment with blocking regent (2% bovine serum albumin in 100 mM Tris-HCl (pH 7.5) and 150 mM NaCl), the sections were incubated with an alkaline phosphatase-conjugated anti-DIG antibody (1:2000; Roche Diagnostics GmbH, Mannheim, Germany) for 18 h at 4 °C. Color development was performed using a DIG detection kit (Roche), following the manufacturer’s protocol, using detection buffer containing nitro-blue tetrazolium chloride and 5-bromo-4-chloro-3′-indolylphosphatase. After terminating the reaction with Tris-EDTA, sections were attached to gelatin-coated slides and air-dried. Finally, sections were washed with 100% methanol, dehydrated with 100% ethanol, cleared with xylene, and then mounted with Entellan New (Fujifilm, Osaka, Japan).

### 4.3. In Situ Hybridization Chain Reaction and Immunofluorescence Staining

In situ HCR was performed using an ISH palette (Nepa Gene Co., Ltd., Chiba, Japan). The DNA probes using short-hairpin DNAs are shown in Table 1. Oligo DNA probes were designed by Nepa Gene and synthesized as standard desalted oligos by Integrated DNA Technologies. Furthermore, SaraFluor 488^TM^-S23, ATTO550-S73, and ATTO647N-S10 were used as hairpin DNA amplifiers (Nepa Gene Co., Ltd.). The sections were thawed to adhere to MAS-coated slides (Matsunami Glass Ind., Ltd., Osaka, Japan) and air-dried briefly. The slides were washed with PBS (pH 7.4) and incubated in methanol for 10 min at room temperature (RT). Slides were washed with PBS containing 0.1% Tween20 (PBST) and placed in a pre-warmed humidity control tray. A mixture of probes in Hybridization Buffer (Nepa Gene Co., Ltd.) was pipetted onto each section until fully submerged. Each slide was covered with Parafilm to prevent it from drying. The humidity control tray was placed in an oven for 18 h at 37 °C. After hybridization, the sections were washed thrice with 0.5× SSC containing 0.1% Tween 20 (0.5× SSCT) for 10 min at 37 °C, followed by incubation with a mixture of ISHpalette Short hairpin amplifiers in the humidity-control tray for 2 h at RT. After HCR, slides were washed twice with PBST for 10 min at 37 °C and underwent immunofluorescence staining. The sections were blocked with 5% normal goat serum in PBST and incubated with primary antibody using an anti-TH antibody (1:2000; mouse monoclonal, clone LNC1, MAB318, Sigma Aldrich Japan G.K., Tokyo, Japan) [37] or anti-PV (1:2000; mouse monoclonal, clone PARV-19, P3088, Sigma Aldrich) [38,39] for 18 h at 4 °C. After washing the sections with PBST thrice for 5 min, AlexaFluor405-conjugated anti-mouse IgG was added to the sections and incubated for 1 h at RT. Furthermore, the sections were washed and coverslipped with Fluoromount (K024; Diagnostic BioSystems Inc., Pleasanton, CA, USA). In addition, the sections were visualized using a fluorescence microscope (BZ-X710; Keyence, Osaka, Japan) or a confocal microscope (LSM 780, Carl Zeiss, Jena, Germany).

### 4.4. Immunohistochemical and Immunofluorescence Staining

Animals were anesthetized with a combination anesthetic (0.4 mg/kg of medetomidine, 2.0 mg/kg of midazolam, and 2.5 mg/kg of butorphanol), and transcardially perfused with physiological saline, followed by an ice-cold fixative containing 4% paraformaldehyde and 0.2% picric acid in 0.1 M PB (pH 7.4). Sectioning was performed as previously described [16]. Briefly, the brains were postfixed with the fixative overnight at 4 °C, followed by immersion in 30% sucrose for at least 48 h at 4 °C. The tissues were then embedded for cryo-sectioning in Tissue-Tek OCT and sectioned at 30 µm on a Cryostat NX70.

The sections were washed with PBS, immersed in methanol for 10 min, followed by PBST washing twice for 5 min, and then incubated in PBST containing 1% hydrogen peroxide for 30 min at RT. After washing with PBST, sections were incubated with a blocking solution containing 5% normal goat serum (Vector Laboratories, Inc., Burlingame, CA, USA) in PBST for 1 h at RT and then incubated with anti-MC1R antibody (1:300; rabbit polyclonal, GTX108190, GeneTex, Inc., Irvine, CA, USA) [40] for 48 h at 4 °C. After washing with PBST, the sections were incubated with a biotinylated goat anti-rabbit antibody (Vector Laboratories) for 1 h at RT. The sections were then washed and incubated with the avidin–biotin–peroxidase complex (ABC kit; Vector) for 1 h at RT. Sections were subsequently incubated in 50 mM Tris-HCl (pH 7.6) containing 0.05% 3-3-diaminobenzidine tetrahydrochloride (DAB; Dojindo, Kumamoto, Japan) and 0.003% hydrogen peroxide for 10 min at RT. Notably, all sections were adhered to gelatin-coated slides, dehydrated using graded concentrations of ethanol, cleared in xylene, and mounted with Entellan New. Images were acquired using a bright-field light microscope (Olympus AX80, Tokyo, Japan).

Furthermore, to confirm the co-localization of MC1R and Atrn in dopaminergic neurons marked by the TH antibody, parvalbumin neurons marked by the PV-antibody, or GABAergic neurons marked by *VGAT* mRNA, multi-fluorescent labeling was performed. The anti-Atrn antibody (1:100; guinea pig polyclonal, a synthetic peptide comprising amino acids 1403–1416 of the rat attractin sequence; SQQMPIVYKEKSGA; GenBank accession no. BAB21017.1, Supplementary Materials Figure S2) was developed using synthetic peptides. The sections were washed with 0.1 M PBS, immersed in methanol for 10 min, washed with PBST, and then blocked with 5% normal goat serum for 1 h at RT. The sections were incubated with primary antibodies (anti-MC1R, anti-Atrn, and anti-TH or anti-PV) prepared in PBST containing 1% bovine serum albumin for 48 h at 4 °C. After washing with PBST, the sections were treated with Alexa Fluor 405, 488, and 568 secondary antibodies (Supplementary Materials Figure S3) for 1 h at RT. We used a single in situ HCR for *VGAT* mRNA combined with triple-immunofluorescence for MC1R, Atrn, and PV to detect the coexistence of GABAergic neurons. Coverslips were mounted on slides with Fluoromount, and sections were visualized using a fluorescence microscope (BZ-X710, Keyence, Osaka, Japan) or a confocal microscope (LSM 780, Carl Zeiss, Jena, Germany).

### 4.5. Pre-Embedding Immuno-Electron Microscopic Study for MC1R

Sections (30 µm thickness) were cryoprotected with 30% sucrose and flash-frozen in isopentane cryogen to prepare sections for pre-embedding immune-electron microscopic study. Sections were collected in 30% sucrose, washed with 0.1 M PBS, and incubated in 1% hydrogen peroxide for 30 min at RT to inactivate endogenous peroxidase activity. After washing with 0.1 M PBS, they were pretreated with a blocking solution containing 5% normal goat serum for 1 h at RT and then incubated with MC1R antibody for 48 h at 4 °C. After washing with 0.1 M PBS, the sections were incubated in biotinylated anti-rabbit IgG for 1 h at RT. The sections were then washed and placed in an avidin–biotin–peroxidase complex for 1 h at RT. The immunoreaction was then visualized by reacting with 50 mM Tris-HCl buffer (pH 7.6) containing DAB and 0.003% hydrogen peroxide for 10 min at RT. After 1% Osmium tetroxide treatment, the sections were dehydrated and embedded in Epon-812 resin (TAAB, Berkshire, UK). Ultrathin sections (70 nm thick) were cut using a Leica EM UCT ultramicrotome (Leica Microsystems, Wetzlar, Germany) and imaged using an electron microscope (IT-800SHL; JEOL, Tokyo, Japan).

## Figures and Tables

**Table 1:** Split-initiator probe sequences.

Probe Name	First Probe	Second Probe
MC1R-1S23	GGGTGGTCGaaCCAGGGTAGGCTGAGGTCTGGGGAA	AACTTCTCGGTTCCTTGCCTCGCCAaaTCGAAGTCGTAT
MC1R-2S23	GGGTGGTCGaaAGACATTTCTAAACTTGACACCTCC	ATGTCTCTCCCCAAGTGGCTCAGGTaaTCGAAGTCGTAT
MC1R-3S23	GGGTGGTCGaaGGAGCTTAGCTTTCTGCTGTTCCCC	CTCACGGAGTCTCACTGCCAGTGTGaaTCGAAGTCGTAT
MC1R-4S23	GGGTGGTCGaaTTCCTGGCTAGGACCCTGAGCTCTA	TAGGCATGGCATAGGCAGTCTAGACaaTCGAAGTCGTAT
MC1R-5S23	GGGTGGTCGaaCCCTGTCTGCCTGGACAGGTCAGCC	ATGCTCCGATGTTCCTTCACACATGaaTCGAAGTCGTAT
MC1R-6S23	GGGTGGTCGaaCAGACCTTCCTCTTTCCATACGGGC	TTGTTGTCAGGGGTCTCTGACATTCaaTCGAAGTCGTAT
MC1R-7S23	GGGTGGTCGaaCTTCCAGCTTCCGACTCAGCTCATG	GGAGGCTGCAGCTCCAGGTGGTATGaaTCGAAGTCGTAT
MC1R-8S23	GGGTGGTCGaaAGAGAACCCAGAAGCCTCTTCGGGG	TGAGAGGTGGTGATGGAAGTGGAGTaaTCGAAGTCGTAT
MC1R-9S23	GGGTGGTCGaaTGTCTGGTTGGTGGCCAGTCCGAGG	GATAGACACATGCAGGCACCAAGACaaTCGAAGTCGTAT
MC1R-10S23	GGGTGGTCGaaCTCACCAGCCCCAGGCTGAGGAAGA	ACCACAACCAACACGTTCTCCACCAaaTCGAAGTCGTAT
MC1R-11S23	GGGTGGTCGaaCCATCAGGTCAGACAGGGCCAGACA	TGGTCTCCAGCACGATGCTCACACTaaTCGAAGTCGTAT
MC1R-12S23	GGGTGGTCGaaTGTCCAGCTGCTGCACCAGAGCCGC	AGCCACAGATGAGCACGTCGATGACaaTCGAAGTCGTAT
MC1R-13S23	GGGTGGTCGaaAGGAAGCAGAGACTGGACACCATGG	ATGTAGCGGTCTATGGCGATGACACaaTCGAAGTCGTAT
MC1R-14S23	GGGTGGTCGaaTGCTGACCACCCAGATGCCCACGAC	AGGTGATGAAGAGGGTGCTGGAGACaaTCGAAGTCGTAT
MC1R-15S23	GGGTGGTCGaaAGTGACGAGGCAGAGCAGGACGGCC	CATGAGTGCCAGCATGGCTAGGAAGaaTCGAAGTCGTAT
MC1R-16S23	GGGTGGTCGaaAGAGCATGTGGACATACAGAATTGC	TGCCCTGAGCATGCTGGCACGCTCGaaTCGAAGTCGTAT
MC1R-17S23	GGGTGGTCGaaGCAAGAGGTGCAGGAAGAAAGGGGC	TGGGGTGCTGAGGGCAGAGAACGATaaTCGAAGTCGTAT
MC1R-18S23	GGGTGGTCGaaCTGGCCGTGAGACCGAAAGCCACTT	TATCACTGTCACCCTCTGCCCAGCGaaTCGAAGTCGTAT
MC1R-19S23	GGGTGGTCGaaTCTCAACAGGATGTGGGCCACTGGA	AGATCAGGAAGGGATGAGTACCTGTaaTCGAAGTCGTAT
MC1R-20S23	GGGTGGTCGaaATCCAGTCCACCCTTAGACAAAGGG	GCACATTGTGTTTCTATTTTAAAGTaaTCGAAGTCGTAT
Atrn-1S73	CGGTCAGGTaaAGAAGATCCAGTTAGTCTGAAGCGG	ATTCCCAGGTCCATCTGTTACAAATaaGGCTAGTATGGA
Atrn-2S73	CGGTCAGGTaaCTTCAATGAGCCATGTGCATTTCGT	GAAGTCTCATTATCTTATTTGGCTGaaGGCTAGTATGGA
Atrn-3S73	CGGTCAGGTaaAGATGCCTCGGTGAGGAAAGCCACA	AGCACCCTCTGGTGTCACTTGCGTTaaGGCTAGTATGGA
Atrn-4S73	CGGTCAGGTaaGGACCCTGCCAGTGAGGGAAGCAGG	TTAGCTGGCACAGGAATTGAACATCaaGGCTAGTATGGA
Atrn-5S73	CGGTCAGGTaaTCAGAATATTCTTCTCGAGTCCAAA	TTATGAGAGGCTCTGGGAAGCTTTAaaGGCTAGTATGGA
Atrn-6S73	CGGTCAGGTaaATCTGAATGGTTGAACATATACCCG	CAGGTCATACGCTAAAACCATGCTGaaGGCTAGTATGGA
Atrn-7S73	CGGTCAGGTaaTTAGTGAAAGCCATTCCCTAGAAGC	TTACAACCACACTGTTCACAGAATGaaGGCTAGTATGGA
Atrn-8S73	CGGTCAGGTaaTGCATACTGATCCTTAGCTTTGGGG	AACAATGTGTGCTGAGTGTCCAACCaaGGCTAGTATGGA
Atrn-9S73	CGGTCAGGTaaAAGATGACCAACATGACCACACGGC	ATATATCCGTAGAGCGGACAATGACaaGGCTAGTATGGA
Atrn-10S73	CGGTCAGGTaaTACTCCATGTGTTCTTTTCCAAGTC	GCACTAGAGCACCTTGAGTTTGTAAaaGGCTAGTATGGA
Atrn-11S73	CGGTCAGGTaaATCTGTAGAGGTCATCTGCAAGCCG	TGGTCCACATCTGAGTGTCCACATGaaGGCTAGTATGGA
Atrn-12S73	CGGTCAGGTaaTCACAGCTGTATGCAAGTAACGGAA	CGAACACCAGCATGGTTCCACTCACaaGGCTAGTATGGA
Atrn-13S73	CGGTCAGGTaaTGACATCGTGATGGAGCTCAGGTCT	GCAAGACTGCTGAGTGGCCAAATCGaaGGCTAGTATGGA
Atrn-14S73	CGGTCAGGTaaACTGAGGAGGAGGCTGTTGAAGCCG	CTGCTCCGAGGTGAAGACTAAGACGaaGGCTAGTATGGA
Atrn-15S73	CGGTCAGGTaaGGTCCTGCTGCCACACAAGCAGCTT	TGTGTGTCCCACAGACACCGGATGCaaGGCTAGTATGGA
Atrn-16S73	CGGTCAGGTaaCTCCCAGGAGGTACATCGAGACGAC	CTTTTCTGCTTGTTCTTCAGTTGCCaaGGCTAGTATGGA
Atrn-17S73	CGGTCAGGTaaTTCTTTTAGAGAAACATTCTGATTT	GCTGGTCACATCTGTCATGGTCAAGaaGGCTAGTATGGA
Atrn-18S73	CGGTCAGGTaaGTGGTTCACAGGGACACAGTGATCA	AATGGAGATCTGGCCTTCTGTGCAGaaGGCTAGTATGGA
Atrn-19S73	CGGTCAGGTaaGTAGTACATGGGGTTATCCTTGGGG	GCTCCTGCAGCTGGTTTTCTTATTGaaGGCTAGTATGGA
Atrn-20S73	CGGTCAGGTaaACTGGCAATTCTGATCTAGGGCACA	CGATGCACTCTTGATTTCGAGGTTCaaGGCTAGTATGGA
VGAT-1S10	CGTCGGATGaaTGGTCAGCTTGCTGCGGAGCAGGGT	ACTTGTTGGACACAGAGGTGGCCACaaAGCCCATTAGAT
VGAT-2S10	CGTCGGATGaaTGCTCAAAGTCGAGATCGTCGCAGT	AGGATGTCCATCTGCAGGCCCTGGCaaAGCCCATTAGAT
VGAT-3S10	CGTCGGATGaaATGGCGTTTGTCACGTTCCAGCCCG	GGTAGACCCAGCACGAACATGCCCTaaAGCCCATTAGAT
VGAT-4S10	CGTCGGATGaaTCTTGCCGGTGTAGCAGCACACCAC	TCTCCTCGTACAGGCACGCGATGAGaaAGCCCATTAGAT
VGAT-5S10	CGTCGGATGaaACGCGCACCACCTCACCATCTTCGT	TTAGCTATGGCCACATACGAGTCCCaaAGCCCATTAGAT
VGAT-6S10	CGTCGGATGaaGATCTGGGCCACATTGACCACGCGG	CAAGATACACGTCATCACCAGCTCGaaAGCCCATTAGAT
VGAT-7S10	CGTCGGATGaaAGGAAGGCGCAGGGCAGCAGCACCG	AACTTGGACACGGCCTTGAGATTCTaaAGCCCATTAGAT
VGAT-8S10	CGTCGGATGaaGTGGGCCAGCGTGCACAGCAGACTG	GGCGATGACCAGGATGTTGATGACGaaAGCCCATTAGAT
VGAT-9S10	CGTCGGATGaaCCTTCTCCCAGGCCCAGTCACGCGC	ACTTCTTGACGTCGATGTAGAACTTaaAGCCCATTAGAT
VGAT-10S10	CGTCGGATGaaACGATGATGCCGATGGAGATAGGAA	AGGAAGATCTGCGACGTGTAGCTGAaaAGCCCATTAGAT
VGAT-11S10	CGTCGGATGaaCTGCATGTTGCCTTCGAGCGAGGGC	CATCATGCAGTGGAATTCGCTGGGCaaAGCCCATTAGAT
VGAT-12S10	CGTCGGATGaaTTGGCCACCAGGAAGATGTTGACCA	AAGGGCAACGGGTAGGACAGCAGCGaaAGCCCATTAGAT
VGAT-13S10	CGTCGGATGaaAGACTTCTCCAGCACTTCGACGGCC	GAAGGCACGACTGCCTTCCTGGAAGaaAGCCCATTAGAT
VGAT-14S10	CGTCGGATGaaGCCATGAGCAGCGTGAAGACCACCA	AGCAGCGCGAAGTGTGGCACGTAGAaaAGCCCATTAGAT
VGAT-15S10	CGTCGGATGaaAAGAGGCTGGGCAGCAGGAAGCAGA	AGCTTGCGCCAGAGAAGACGCAAGTaaAGCCCATTAGAT
VGAT-16S10	CGTCGGATGaaTCGAGTGAATGCACGAAGCCGGACA	TTGGTTCGGTAGGCCTCGATGAGGCaaAGCCCATTAGAT

## Data Availability

All data are available from the corresponding author upon reasonable request.

## Supplementary Materials

The following supporting information can be downloaded at https://www.mdpi.com/article/10.3390/ijms26010236/s1.
