# Supplementary material for: Localization of Melanocortin 1 Receptor in the Substantia Nigra
Source: Int J Mol Sci. 2024 Dec 30;26(1):236. doi: 10.3390/ijms26010236 (PMC11720287; doi:10.3390/ijms26010236)
Supplement: Supplementary file 1 [file ijms-26-00236-s001.zip › ijms-3372931 supplement Material_1.pdf]

Figure S1

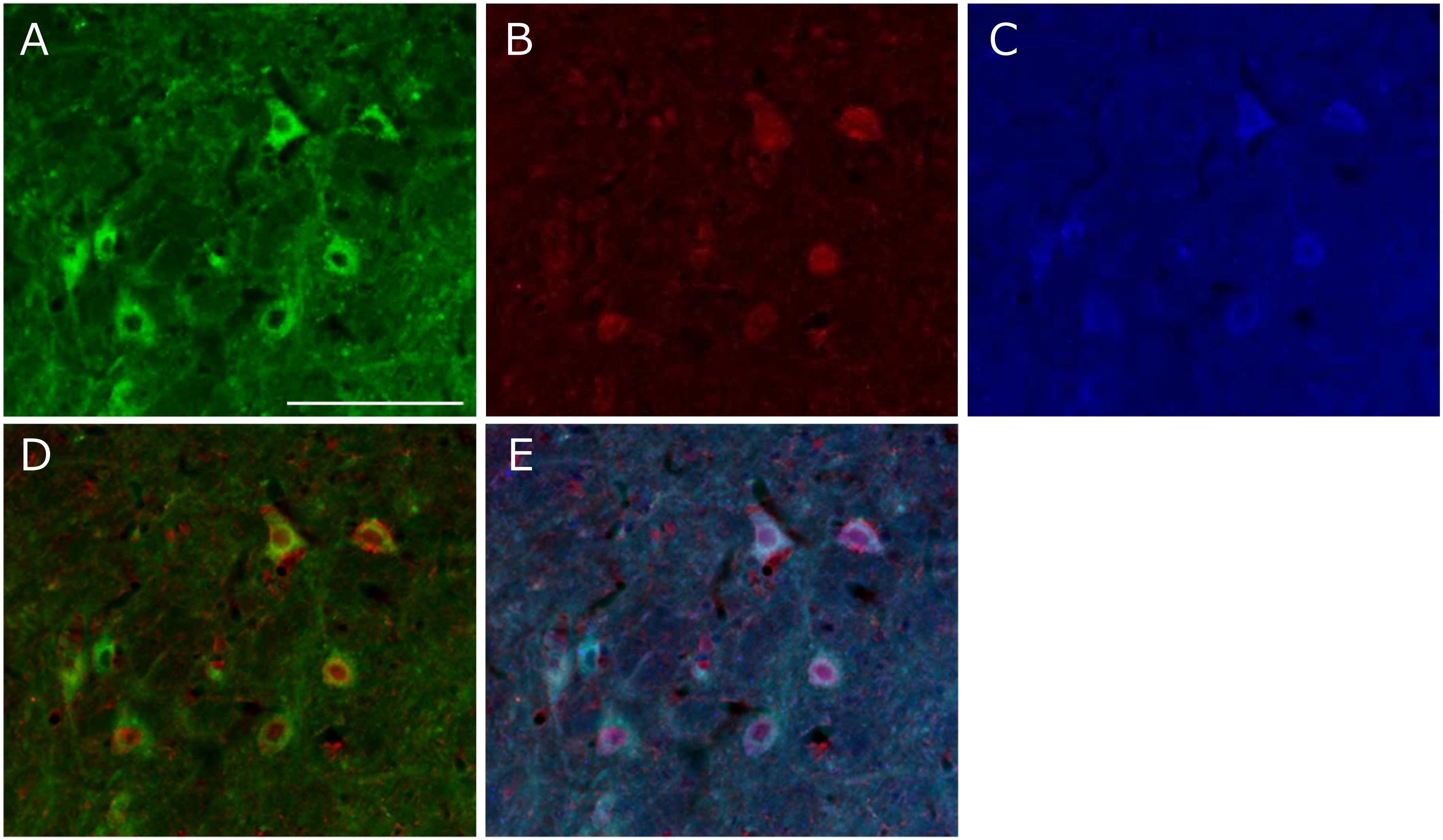

The presence of *MC1R* mRNA expression and its immunopositivity in the same PV neurons of the SNR. Epifluorescence images showing (A) *MC1R* mRNA expression, (B) MC1R immunoreactivity, (C) PV immunoreactivity, (D) the merge of *MC1R* mRNA expression (green) and MC1R immunoreactivity (red), and (E) the merge of *MC1R* mRNA expression (green), MC1R immunoreactivity (red), and PV immunoreactivity (blue). Scale bar: 100 μm
