# Supplementary material for: Localization of Melanocortin 1 Receptor in the Substantia Nigra
Source: Int J Mol Sci. 2024 Dec 30;26(1):236. doi: 10.3390/ijms26010236 (PMC11720287; doi:10.3390/ijms26010236)
Supplement: Supplementary file 1 [file ijms-26-00236-s001.zip › ijms-3372931 supplement Material_2.pdf]

Figure S2

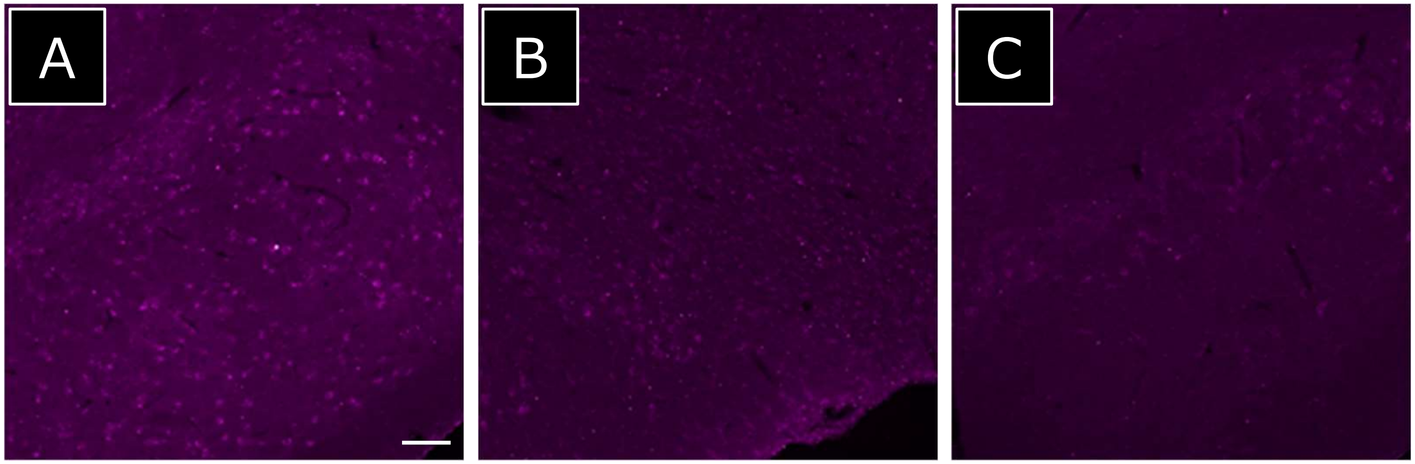

The specificity of the Atrn antibody used in the immunofluorescence staining of this experiment. Epifluorescence images for Atrn (magenta) in the SN of (A) a SD rat and (B) a Zitter rat, which is an *Attractin* mutant rat. (C) The image in the SN of a SD rat using only the secondary antibody (Alexa568-conjugated guinea pig IgG), without the primary antibody. Scale bar: 100 μm
