# Supplementary material for: Localization of Melanocortin 1 Receptor in the Substantia Nigra
Source: Int J Mol Sci. 2024 Dec 30;26(1):236. doi: 10.3390/ijms26010236 (PMC11720287; doi:10.3390/ijms26010236)
Supplement: Supplementary file 1 [file ijms-26-00236-s001.zip › ijms-3372931 supplement Material_3.pdf]

Figure S3

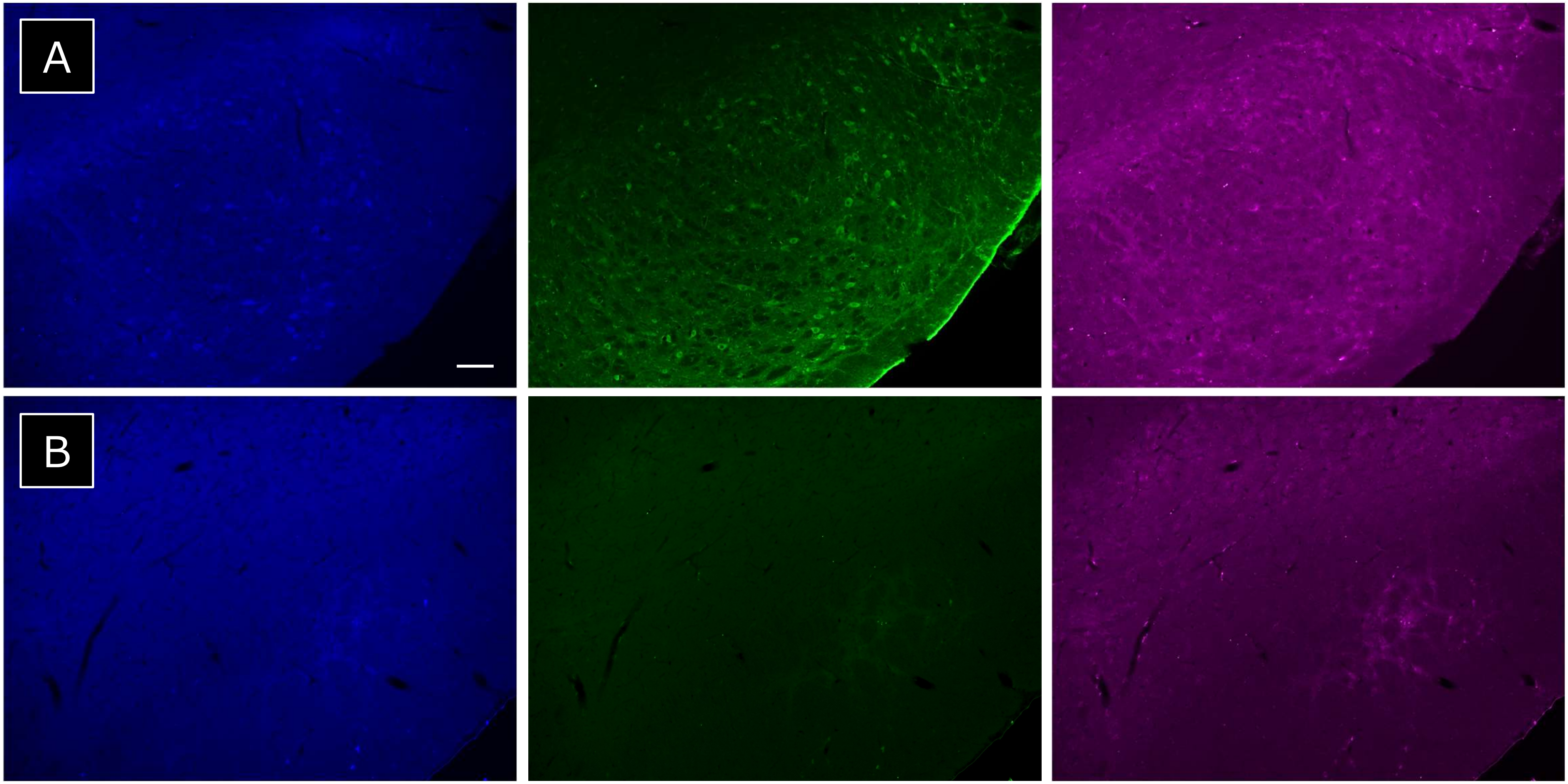

The immunofluorescence reaction disappeared in the absence of primary antibodies. Epifluorescence images (A) with primary antibodies for PV (blue), MC1R (green), and Atrn (magenta), (B) without primary antibodies and with secondary antibodies for Alexa405-mouse IgG (blue), Alexa488-rabbit IgG (green), and Alexa568-guineapig IgG (magenta) in the SN of a SD rat. Scale bar: 100 μm
